# Sputum *Moraxella catarrhalis* strains exhibit diversity within and between COPD subjects

**DOI:** 10.2147/COPD.S180961

**Published:** 2018-11-08

**Authors:** Leena M George, Richard D Haigh, Vijay Mistry, Koirobi Haldar, Michael R Barer, Marco R Oggioni, Christopher E Brightling

**Affiliations:** 1 Department of Infection, Immunity and Inflammation, University of Leicester, Leicester, UK, ceb17@le.ac.uk; 2 Department of Genetics, University of Leicester, Leicester, UK

**Keywords:** COPD, *Moraxella catarrhalis*, strain change, exacerbation

## Abstract

**Purpose:**

*Moraxella catarrhalis* is implicated in the pathogenesis of some COPD exacerbations. We sought to investigate whether the *M. catarrhalis* strain is variable between COPD subjects; that an exacerbation is associated with acquisition of a new strain and that certain strains are more commonly associated with exacerbations.

**Patients and methods:**

Sputum samples were collected at stable and exacerbation visits from COPD subjects from a single center as part of the COPDMAP consortium. Samples identified as *M. catarrhalis* positive by qPCR were recultured in liquid cultures grown to extract genomic DNA; underwent Illumina MiSeq and bacterial genome sequences were de novo assembled and Multi Locus Sequence Type (MLST) was determined.

**Results:**

Thirty-five samples were obtained from 18 subjects. These included 13 stable and 22 exacerbation samples. The diversity between samples was very large with 25 different *M. catarrhalis* MLSTs being identified out of the 35 samples of which 12 MSLTs have not been described previously. Change and persistence of *M. catarrhalis* strain were observed between stable visits, from stable to exacerbation and vice-a-versa, and between exacerbation visits.

**Conclusion:**

Sputum *M. catarrhalis* strains exhibit marked diversity within and between COPD subjects. Acquisition of a new strain is common between stable and exacerbation events such that no strain is specifically associated with an exacerbation.

## Introduction

COPD is characterized by irreversible airflow obstruction and airway inflammation.^1^ Bacteria are commonly identified in stable state and at exacerbations with increased bacterial load and dysbiosis associated with exacerbations.^2^–^9^ The most common pathogens identified are *Haemophilus influenzae* and *Streptococcus pneumoniae* in both stable disease and exacerbations, but several other pathogens are identified including *Moraxella catarrhalis*.^2^–^8^ Using molecular approaches *M. catarrhalis* in sputum samples typically has a prevalence of 10%–20% in exacerbation samples, whereas in stable disease it is more unusual with a prevalence of 5%–10%.^2^,^6^ Even though *M. catarrhalis* is not the most prevalent bacteria in COPD sputum samples at exacerbation the abundance of this bacterium increases consistently in a subgroup with an associated increase in sputum inflammatory mediators.^4^ This underscores the importance of *M. catarrhalis* in a minority of COPD exacerbations. Previous studies have suggested that these events are a consequence of acquisition of a new *M. catarrhalis* strain;^9^,^10^ however, this remains to be determined as these studies did not apply next-generation sequencing approaches. We hypothesized that *M. catarrhalis* strain is variable between COPD subjects; that an exacerbation is associated with acquisition of a new strain and that certain strains are more commonly associated with exacerbations.

## Materials and methods

Spontaneous sputum samples were collected at stable and exacerbation visits from COPD subjects from a single center, the University of Leicester, as part of the COPDMAP consortium (www.copdmap.org; NCT01620645).^6^ Samples with high squamous cell contamination (>50%)^11^ were not included in this report, but only represented a small proportion of the study as a whole and assessable sputum was available in >90% of visits.^6^ Samples collected at exacerbation were immediately before initiation of systemic corticosteroids or antibiotics. All subjects gave informed written consent, and the study was approved by the local ethics committee (Leicestershire and Northamptonshire ethics committee). Sputum samples were processed to generate sputum cytospins and a sputum plug was processed with the mucolytic DTT and glass beads. The homogenized sample was used for both routine culture and molecular identification. Sputum samples were cultured on blood agar plates and cultures of single colonies were stored in freezing broth (brain heart infusion broth +10% glycerol) at −70°C. In 12 samples, two colonies were stored to assess within-sample variability. Bacterial genomic DNA was extracted from sputum samples using the Qiagen DNA Mini kit (Qiagen, CA, USA) as per manufacturer’s pr5otocol. The V4 hypervariable region of the 16S rRNA gene was PCR amplified and sequenced using multiplex libraries on the Illumina MiSeq platform as described previously. Sequencing reads were processed using QIIME pipeline version 1.9. Samples from subjects that were identified as positive for *M. catarrhalis* using standard culture from at least one visit, and for which there were stored culture samples, were recultured in liquid cultures grown to extract genomic DNA to make Nextera XT sequencing libraries for analysis on Illumina MiSeq. Bacterial genome sequences were de novo assembled using SPAdes^12^ and an online Multi Locus Sequence Type (MLST) check for *M. catarrhalis* was undertaken at the Center for Genomic Epidemiology^13^ (CGE; https://cge.cbs.dtu.dk//services/MLST/). Genome sequences that could not be typed at CGE were submitted to Enterobase^14^ (http://enterobase.warwick.ac.uk/) to be allocated to novel *M. catarrhalis* MLST groups. Assembled genome sequences were annotated using prokka^15^ and pangenome analysis was performed using Roary.^16^ Cladograms were drawn using FigTree (http://tree.bio.ed.ac.uk/software/figtree). Descriptive statistical analysis was undertaken using PRISM (graphpad, La Jolla, CA, USA).

## Results

A total of 35 samples were obtained from 18 subjects. These included 13 stable and 22 exacerbation samples. Of these 18 subjects, six were women and 12 were men. The mean (standard error of the mean) age was 69 (7) years, smoking pack years 50 (26), post-bronchodilator FEV_1_ % predicted 52 (14), FEV_1_/FVC % 49 (9), and SGRQ total score 52 (20). All subjects were treated with triple therapy inhaled corticosteroids (ICS), in combination with a long-acting muscarinic antagonist and long-acting beta-agonist (LABA) except for one subject that received ICS-LABA. The median (IQR) sputum eosinophil and neutrophil count were 0.75 (2.5) % and 79 (46) %, respectively. The prevalence of *M. catarrhalis* was higher in the selected subgroup than the whole COPDMAP cohort (14% vs 5% of the total microbiome; *P*<0.001). The prevalence of *H. influenzae* and *S. pneumoniae* was similar in the subgroup and full cohort (25% vs 25%) and (3% vs 4%), respectively.

**Figure 1:**
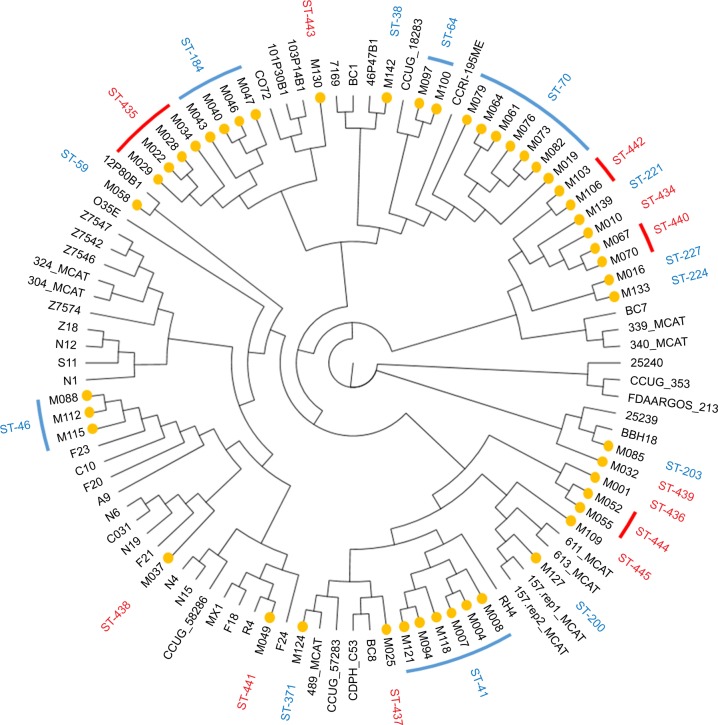
Cladogram of 100 *Moraxella catarrhalis* genomes based upon their accessory genomes as identified by Roary pangenome analysis. **Notes:** Genomes from strains identified in this work are highlighted with a yellow symbol. MLST groupings are shown in the outer ring with previously known MLST in blue and novel MLST in red. **Abbreviation:** MLST, Multi Locus Sequence Type.

**Figure 2:**
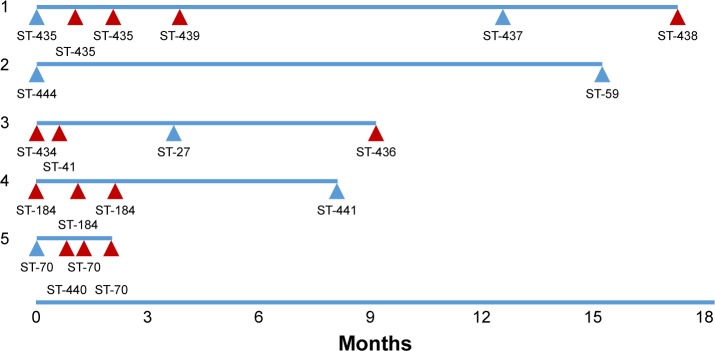
*Moraxella catarrhalis* strain change with time. **Notes:** Timelines are shown for the five subjects in whom data were available from more than one visit including at least one sample in stable state; each triangle depicts a sample (blue = stable visit, red = exacerbation) with the MLST of the strain identified for each visit.

Forty-eight individual *M. catarrhalis* colonies were sequenced from the 35 samples (23 samples had a single colony, 11 samples had two colonies, and one sample had three colonies sequenced). The diversity between samples was very large with 25 different *M. catarrhalis* MLST types (12 of which have not been described previously) identified from the 35 samples (all 48 genome sequences have been deposited in NCBI GenBank as BioProject PRJNA488991). Of the 12 samples in which multiple colonies were available for sequencing, only one contained two different strains as defined by MLST type. A phylogenetic analysis comparing the 48 strains sequenced in this work with the 52 *M. catarrhalis* genomes currently available from GenBank is shown in Figure 1; the cladogram, which is based upon accessory genome content, shows that all of the strains identified in this work fell into clades alongside previously sequenced genomes. Changes in *M. catarrhalis* strain were observed between stable visits, from stable to exacerbation and vice-a-versa, and between exacerbation visits. Persistence of an *M. catarrhalis* strain was also observed from stable to exacerbation and between exacerbation visits. Between subjects, only three of the 25 MLST groups were shared (ST-41, ST-46, and ST-70), whereas 22 were unique within subjects. For the five subjects with more than one sample and at least one stable sample taken, the changes in the MLST of *M. catarrhalis* strains identified over time are shown in Figure 2.

## Discussion

We found that sputum *M. catarrhalis* strains exhibit marked phylogenetic diversity within and between COPD subjects. As a consequence of this diversity the acquisition of a new strain was a common event and occurred between stable and exacerbation visits as did persistence of a strain. No MLST or strain was specifically associated with an exacerbation event.

Our findings extend previous studies that have explored *M. catarrhalis* strain change^10^ to now include detailed phylogenetic typing. We report that the diversity of *M. catarrhalis* strains is very large; furthermore, we were unable to determine if acquisition of new strain was important for the onset of an exacerbation because new acquisition was common to most samples irrespective of whether a new stable or new exacerbation visit. Although we cannot discount the possibility that specific *M. catarrhalis* strains are associated with an exacerbation, the number of different MLST types identified here within such a relatively small sample size makes this unlikely.

Our study has a number of limitations. Despite the sample size being the largest to date for genomic sequencing of *M. catarrhalis* cultures from a large observational study, the number of samples and subjects remains modest and larger longitudinal studies are required to determine whether acquisition of a new strain is associated with exacerbations, other clinical features, and inflammatory profiles. It would also be important to test multiple isolated colonies from all samples in order to assess for simultaneous carriage of more than one strain. However, in samples where more than one colony was sequenced, simultaneous carriage was rare. The study relied upon sputum sampling with the inherent challenges of consistent sampling in large populations and oral contamination. The success rate for sputum samples was very high in this group and salivary contamination low;^6^,^11^ therefore, we are confident that these data do reflect lower airway samples.

## Conclusion

In conclusion, sputum *M. catarrhalis* strains are phylogenetically diverse in COPD and whether acquisition of a new strain is clinically important or if an exacerbation represents an emergent phenomenon due to a number of host–environment interactions remains to be determined.
